# The impact of implementing the women's reproductive rights agenda on climate change

**DOI:** 10.3389/fgwh.2025.1594066

**Published:** 2025-06-26

**Authors:** Marleen Temmerman, Emilie Peeters, Celine Delacroix, Malachi Arunda, Sara Khalid, Claudia Hanson, Samuel Akombeng Ojong

**Affiliations:** ^1^ Centre of Excellence for Women and Child Health, Aga Khan University, Nairobi, Kenya; ^2^ International Centre for Reproductive Health, Ghent University, Ghent, Belgium; ^3^ Interdisciplinary School of Health Sciences, University of Ottawa, Ottawa, ON, Canada; ^4^ Department of Global Public Health, Karolinska Institutet, Stockholm, Sweden; ^5^ Planetary Health Informatics Group at the Centre for Statistics in Medicine, University of Oxford, Oxford, United Kingdom; ^6^ Child Health and Nutrition, UNICEF, Maputo, Mozambique

**Keywords:** women's reproductive rights, Climate resilience, reproductive justice, gender-responsive policy, unmet contraceptive need, sustainable development

## Abstract

The 1994 International Conference on Population and Development (ICPD) established sexual and reproductive health and rights (SRHR) as foundational to sustainable development. Thirty years later, advancing women's reproductive rights (WRR), encompassing agency, decision-making autonomy, and universal access to family planning—remains critical not only for health and gender equity but also for mitigating environmental degradation. By reducing unintended pregnancies and empowering women to align childbearing with personal and ecological capacity, WRR alleviates ecological stressors such as deforestation while enhancing health resilience in climate-vulnerable communities. Yet, despite well-documented linkages between population dynamics and environmental change, contemporary climate policies and funding mechanisms persistently exclude WRR. This oversight undermines the potential of reproductive justice to enhance climate resilience. Additionally, claims that integrating WRR into climate agendas covertly promotes population control or represses women in low- and middle-income countries are fundamentally misleading. Crucially, research is needed to quantify the specific environmental impacts of WRR, underscoring the urgent need for robust global models to predict and validate these co-benefits. Strengthening this evidence base is imperative to inform policies that integrate WRR indicators into climate financing frameworks, ensuring gender-responsive programming. Bridging this gap requires interdisciplinary collaboration to develop metrics that capture WRR's role in reducing resource consumption and enhancing adaptive capacity. Embedding WRR within climate agendas would harmonize reproductive justice with environmental action, unlocking synergies between gender equity, health resilience, and sustainability. Fulfilling the ICPD's vision demands centering WRR in global climate strategies, thereby advancing a just and livable future for all.

## 1 Introduction

Women are disproportionately impacted by climate-sensitive health risks, and their climate vulnerability is further exacerbated by the insufficient realization of the WRR agenda. Despite the 1994 International Conference on Population and Development (ICPD) affirming Women's Reproductive Rights (WRR) as fundamental to sustainable development, persistent gaps in implementation exacerbate vulnerabilities (1, 2). In 2023, an estimated 253 million women globally experienced unmet family planning needs, with nearly one-third residing in Africa—a region acutely susceptible to climate shocks (3). Extreme weather events, such as floods and heatwaves, disrupt healthcare access, escalating maternal mortality, neonatal complications, and gender-based violence (4, 5).

While WRR advancements are proven to enhance women's empowerment, economic participation, and health outcomes (2, 6), their potential to mitigate climate change remains underexplored. Reproductive autonomy optimizes fertility rates, alleviating demographic pressures on finite resources and emissions—a critical yet overlooked feedback loop in climate discourse (7). Concurrently, resilient WRR infrastructure strengthens adaptive capacity by ensuring continuity of care during disasters (8). Despite these dual benefits, global climate frameworks like the Paris Agreement and Green Climate Fund (GCF) neglect WRR integration, perpetuating silos between reproductive justice and environmental governance (9, 10).

Emerging research underscores the urgency of addressing three gaps: (1) mechanisms linking the WRR's agenda to climate change, (2) the magnitude and nature of these impacts, and (3) the identification of vulnerable populations. A structured review of 75 studies reveals regional disparities: 64% focus on Africa and the Western Pacific, masking inequities in South Asia and conflict zones (4). Climate-induced events—extreme heat, droughts, cyclones—correlate with increased HIV prevalence, gender-based violence, and maternal and newborn mortality morbidity (5, 11). However, critical areas remain understudied, including its impact on contraceptive access, abortion services, and reproductive cancers (10, 12, 13).

**Figure 1:**
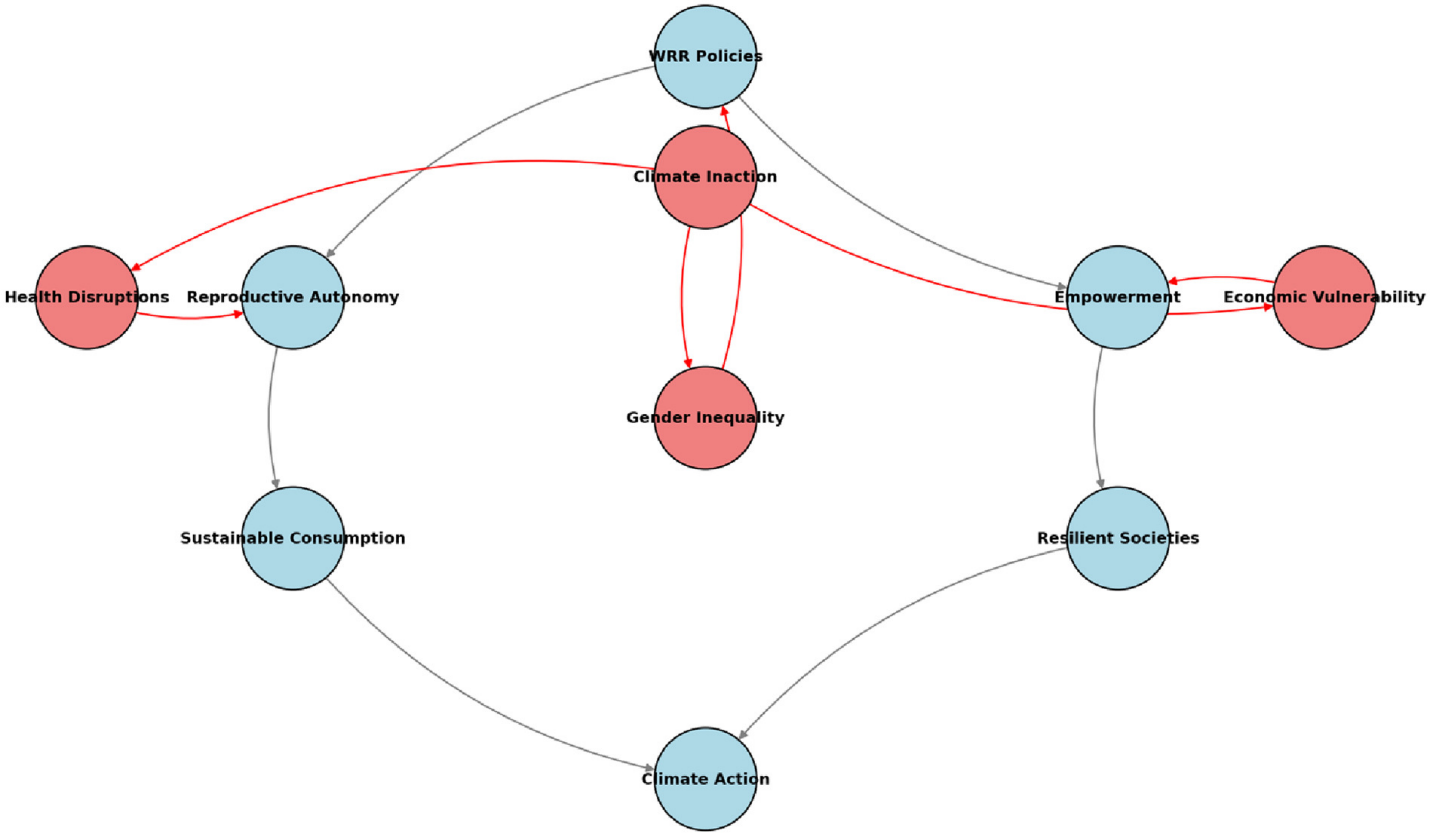
The WRR-climate nexus. The interconnected model presented illustrates the dynamic relationship between WRR and climate. WRR could advance climate resilience (blue), while climate inaction exacerbates gendered vulnerabilities (red) creating a feedback loop that undermines both human rights and planetary health. The model emphasizes health and adaptive co-benefits of WRR, not demographic outcomes alone.

Bridging these gaps demands interdisciplinary research and policy coherence. Quantifying WRR's ecological co-benefits—such as reduced deforestation from slower population growth—requires robust modeling and harmonized climate-SRHR data systems (14). As the Inter-governmental Panel on Climate Change (IPCC) warns of irreversible tipping points, centering reproductive justice in climate governance is essential to safeguarding both human rights and planetary health (15). Integrating WRR into climate resilience strategies is not merely a moral imperative but a pragmatic pathway to achieving SDGs 3 (health), 5 (gender equality), and 13 (climate action). This perspective piece focuses on settings such as sub-Saharan Africa and South Asia where climate shocks intersect with systemic gaps in reproductive healthcare access (see Figure 1). While high-income nations face distinct demographic trends (e.g., aging populations), LMICs bear the dual burden of rapid climate change and unmet contraceptive needs, a nexus demanding urgent policy integration.

## 2 Reproductive rights and climate mitigation: advances, gaps, and future directions

### 2.1 Climate change's impact on women's reproductive health and rights

Climate change exacerbates systemic inequities, directly threatening women's reproductive health and rights through disrupted healthcare access, heightened maternal risks, and entrenched poverty. Extreme weather events—heatwaves, floods, and cyclones—damage health infrastructure, sever supply chains for contraceptives, and displace communities, disproportionately affecting women in low- and middle-income countries (LMICs) (4, 5). For instance, in sub-Saharan Africa, where one out of every three women lack family planning access, climate-induced droughts and floods have led to commodity shortages, increasing unintended pregnancies and maternal mortality (3, 11). Heatwaves alone correlate with a significant rise in preterm births and stillbirths, compounding risks for women already facing limited healthcare access (5).

Also, gender-based violence (GBV) surges during disasters, as displacement and resource scarcity heighten women's vulnerability. In the Western Pacific, cyclones disrupted SRHR services, leading to a rise in adolescent pregnancies and GBV cases (4). Climate-related migration further strains health systems, leaving marginalized groups—rural women, refugees, and indigenous communities—without contraception or maternal care (16). These impacts underscore a critical feedback loop: climate crises erode WRR, while insufficient reproductive autonomy could intensify demographic pressures on ecosystems (7).

Despite these challenges, global climate frameworks like the Paris Agreement and Green Climate Fund (GCF) overlook WRR as a resilience strategy. National Adaptation Plans (NAPs) sparingly mention reproductive health, reflecting systemic silos between environmental and gender equity agendas (17). Prioritizing WRR in climate policy is not merely a health imperative but a prerequisite for breaking this cycle of vulnerability. Critically, these impacts are not merely demographic but humanitarian: climate-induced disruptions to SRHR perpetuate cycles of poverty, gender inequality, and health inequity. Strengthening WRR is thus a prerequisite for breaking these cycles, while ensuring that women's bodily autonomy and health resilience are prioritized alongside environmental sustainability.

### 2.2 Women's leadership, empowerment, and climate policy integration

Advancing women's agency through education and political and economic participation remains a cornerstone of climate resilience. Educated women exhibit lower total fertility rates, greater health literacy, and enhanced leadership in disaster response, directly reducing resource demands and strengthening adaptive capacity (2, 8). In Bangladesh, women-led initiatives established flood-resistant clinics, ensuring continuous access to contraceptives and emergency obstetric care during monsoons (4, 18).

In Thailand, South Korea, Indonesia, Taiwan, and Singapore, family planning programs reduced fertility rates while improving women's economic participation (7). Investing in education and awareness campaigns is essential to inform communities about the intersection of climate change and health, fostering resilience and preparedness.

Empowerment also hinges on legal safeguards, inclusive governance, and policy coherence. Legal frameworks must protect women's participation by incorporating gender quotas in climate governance, as seen in the Pacific Islands' delegation to COP29 (8). Ensuring that international environmental discussions, such as the UNFCCC's Gender Action Plan or COP resolutions, explicitly address WRR is essential to embedding reproductive health as a key pillar of climate resilience (19).

Cross-sectoral policies acknowledging the interrelated nature of gender equity, reproductive rights, population dynamics, and environmental sustainability goals must be developed (19). Also, responses to anthropogenic environmental destruction must integrate population dynamics as a central and transversal component. These policies must be rooted in equity, recognizing that regions like sub-Saharan Africa—which faces rapid population growth, high unmet contraceptive needs, and disproportionate climate impacts—require tailored strategies (20). International frameworks like the Paris Agreement should mandate gender audits of climate finance to ensure investments address both emissions reduction and reproductive justice (10). By institutionalizing these linkages, stakeholders can dismantle silos and foster synergies between SDG 5 (gender equality) and SDG 13 (climate action).

### 2.3 Research and data deficits and imperatives

Critical gaps persist in quantifying WRR's environmental benefits and guiding evidence-based policies. While 64% of SRHR-climate studies focus on Africa and the Western Pacific, South Asia and conflict zones remain underrepresented, masking disparities in abortion access and reproductive cancers (4, 10). No global models estimate the carbon savings of closing the contraceptive access gap, despite evidence that voluntary family planning could impactfully reduce emissions by 2,100 (7). Due to the complexity and scale of leveraging environmental linked with granular health data, advanced analytics will be required. This can benefit from cutting-edge earth observation techniques combined with data fusion and deep learning models to map and model multi-modality, multi-domain environmental measurements alongside health indicators (12, 14, 21–23).

However, sub-Saharan Africa's dual burden of climate vulnerability and unmet family planning needs makes it a critical focus for interdisciplinary research*.* The perspectives of sub-Saharan Africans on population dynamics and climate are uniquely relevant, as this region has experienced the fastest population growth since the 1980s, is projected to almost double in size by 2050, and has the highest proportion of both unintended pregnancies and unmet need for family planning (20). At the same time, despite contributing relatively little to global greenhouse gas emissions, sub-Saharan Africa is disproportionately vulnerable to the impacts of climate change (24).

Bridging the evidence gap between WRR and climate outcomes necessitates interdisciplinary collaboration and advanced data systems. Satellite-based monitoring of heatwaves, paired with real-time contraceptive access metrics, could identify SRHR “hotspots” and prioritize interventions (14). AI-driven platforms, like those predicting malaria outbreaks in South Asia using Earth Observation data, offer blueprints for modeling WRR-climate linkages (12). Also, predictive analytics could quantify how closing the unmet need for family planning—currently affecting 253 million women—might reduce emissions by 2,100 (7). However, funding barriers persist as climate research grants systematically appear to ignore gender-health intersections (10).

A *Global Climate-Reproductive Health Research Alliance* could harmonize datasets across agencies like WHO and the World Meteorological Organization (WMO) enabling real-time mapping of climate risks to SRHR access and predictive analytics for policy decisions (23). Pilot studies in climate-vulnerable regions—such as assessing the impact of heat-resistant clinics on maternal outcomes—could validate scalable solutions (5). Concurrently, embedding SRHR indicators in Green Climate Fund (GCF) criteria and mandating gender audits of climate finance could ensure accountability (10).

### 2.4 Mainstreaming women's reproductive rights in climate finance

Advancing women's reproductive rights (WRR) within climate action demands a radical reorientation of financial mechanisms to prioritize gender-responsive investments. Despite the proven intersection of SRHR and environmental sustainability, less than 1% of global climate finance targets health systems, let alone reproductive healthcare (10). A paradigm shift requires embedding SRHR indicators into climate funding criteria, such as the Green Climate Fund (GCF), to ensure equitable resource allocation. For instance, mandating gender audits of climate finance flows would track equity outcomes and hold institutions accountable (10).

Establishing a dedicated global financing mechanism for SRHR-climate resilience—via multi-actor partnerships involving the Global Environment Facility (GEF) and UNFPA—could channel resources toward climate-resilient, innovative SRHR mobile health units, and telehealth platforms in vulnerable regions (4). The Philippines' typhoon recovery programs allocate resources from adaptation funds to women-led health initiatives, demonstrating the efficacy of targeted financing (16).

Moreover, blended finance models—combining public grants with private investments—can scale innovations like solar-powered clinics in sub-Saharan Africa, where energy shortages disrupt health services including for SRHR (25, 26). Prioritizing SRHR in climate finance should not only seek to align SDGs 3 (health), 5 (gender equality), and 13 (climate action). It must acknowledge both the disproportionate role of production and consumption – primary driven by the Global North – and the population growth complex– predominantly associated with the Global South, as a driver of environmental degradation (15, 20).

## 3 Call to action

Critically, this is not a call for population control but for implementing the women's reproductive rights agenda, and women's decision power as a dual pillar of health equity and climate resilience (Figure 1). Reproductive justice is and should be recognized as a cornerstone of climate resilience. Climate policies influenced by the development and environmental community frameworks often treat population dynamics as immutable, failing to recognize their susceptibility to changes driven by advancements in reproductive rights, gender equity, and education. They further prioritize gender representation while lacking substantive integration of WRR. This risks perpetuating cycles of disempowerment and environmental decline (8, 27).

Transformative change for environmental sustainability requires documenting needs and opportunities from a diverse array of stakeholder perspectives, recognizing that human-nature relationships are shaped by culture and knowledge (28). If women are allowed to exercise agency over fertility —ensuring access to contraception, maternal care, and abortion— they will align childbearing with personal and ecological capacity (2, 4).

Previous critiques framing the integration of women's reproductive rights (WRR) into climate agendas as covert repression or overt population control in LMICs misrepresent the principles underpinning reproductive justice (29, 30). However, we seek to promote and uphold the fundamental right of every woman to autonomous reproductive decision-making, free from governmental, political, religious, cultural, or societal interference. Upholding reproductive rights demonstrably empowers women, enhancing their health, education, and socioeconomic agency, and advancing gender equality and sustainable development (7, 18, 31). Furthermore reproductive autonomy could strengthen women's resilience to climate impacts and contribute to environmental sustainability, decisively refuting claims of a hidden or prejudiced population-control agenda.

Within the WRR- Climate Nexus, policy integration—demands dismantling silos between reproductive justice and environmental governance. Gender-responsive climate laws, which mandate SRHR protections in environmental strategies, could reduce mortality and morbidity during disasters while curbing the impact or drivers of climate inaction (17). Similarly, the Green Climate Fund (GCF) could amplify impact by embedding SRHR indicators into funding criteria, ensuring sustainable and innovative investments in climate-resilient SRHR programs (10).

As the world seeks to avert irreversible tipping points, the ICPD-94 agenda remains a blueprint for a sustainable future. However, its potential is unrealized without explicit integration into climate frameworks. The imperative to tackle the “WRR-Climate Nexus” has never been clearer because reproductive justice, as envisioned by ICPD94, transcends access to healthcare. By centering WRR in climate finance, research, and governance, stakeholders can disrupt harmful feedback loops. When women exercise agency over fertility via accessible contraception, maternal care, and education, they not only improve health outcomes but also foster communities better equipped to adapt to climate change.

## Data Availability

The original contributions presented in the study are included in the article/Supplementary Material, further inquiries can be directed to the corresponding author.
